# Comparative Analysis of Surgical Approaches for Distal Biceps Tendon Rupture: Single-Incision Technique versus Double-Incision Technique

**DOI:** 10.3390/jcm12196423

**Published:** 2023-10-09

**Authors:** Bogdan Hogea, Jenel-Marian Patrascu, Simona-Alina Abu-Awwad, Madalina-Ianca Suba, Andrei Bolovan, Anca Gabriela Stoianov, Ahmed Abu-Awwad

**Affiliations:** 1 Department XV, Discipline of Orthopedics-Traumatology, “Victor Babes” University of Medicine and Pharmacy, Eftimie Murgu Square, No. 2, 300041 Timisoara, Romania; hogea.bogdan@umft.ro (B.H.); jenelmarianp@yahoo.com (J.-M.P.J.); anca.stoianov@umft.ro (A.G.S.); ahm.abuawwad@umft.ro (A.A.-A.); 2 “Pius Brinzeu” Emergency Clinical County Hospital, Bld Liviu Rebreanu, No. 156, 300723 Timisoara, Romania; andrei.bolovan@umft.ro; 3 Research Center University Professor Doctor Teodor Șora, “Victor Babes” University of Medicine and Pharmacy, Eftimie Murgu Square, No. 2, 300041 Timisoara, Romania; 4 Department XII, Discipline of Obstetrics and Gynecology, “Victor Babes” University of Medicine and Pharmacy, Eftimie Murgu Square, No. 2, 300041 Timisoara, Romania; 5 Doctoral School, “Victor Babes” University of Medicine and Pharmacy, Eftimie Murgu Square, No. 2, 300041 Timisoara, Romania; madalina.suba@umft.ro; 6 Dr. Victor Babeș Infectious Diseases and Pneumophthisiology Hospital Timisoara, 300310 Timisoara, Romania

**Keywords:** distal biceps tendon rupture, surgical approaches, comparative analysis, functional outcomes, evidence-based practice

## Abstract

*Background:* This study aimed to compare the outcomes of the single-incision technique with a distal biceps repair system versus the modified double-incision technique, specifically the Morrey-modified approach, Krackow sutures, and drill holes, for the management of acute and chronic distal biceps tendon rupture. The study was conducted at the Orthopedic-Traumatology Clinic II of SCJUPBT Timisoara, Romania, between 2014 and 2022. *Methods:* A total of sixty-nine patients with acute distal biceps tendon rupture and five patients with chronic distal tendon rupture were included in the study. Forty-eight cases underwent the single-incision technique with the distal biceps repair system, while twenty-four patients were treated with the modified double-incision technique. *Results:* Both surgical techniques demonstrated favorable outcomes in terms of successful tendon repair and functional restoration. However, the single-incision technique exhibited slightly superior results in terms of patient satisfaction, range of motion, and postoperative rehabilitation. The modified double-incision technique showed comparable outcomes but had a higher incidence of complications, particularly related to wound healing. *Conclusion:* The single-incision technique with the distal biceps repair system and the modified double-incision technique, including the Morrey-modified approach, Krackow sutures, and drill holes, are effective surgical approaches for the management of distal biceps tendon rupture. The single-incision technique yielded better functional outcomes and patient satisfaction, while the modified double-incision technique had a higher risk of complications. Further research with larger sample sizes and longer follow-up periods is necessary to validate these findings and determine the most appropriate surgical approach for distal biceps tendon rupture.

## 1. Introduction

Distal biceps tendon rupture is an uncommon but debilitating injury that can significantly impair upper limb function and strength [1,2,3]. Prompt and appropriate surgical intervention is crucial to restore optimal muscle function and enhance patient outcomes. Various surgical approaches have been proposed for the management of distal biceps tendon rupture, including the single-incision technique and the double-incision technique [3,4,5]. The single-incision technique involves a minimally invasive approach with a single incision, while the double-incision technique utilizes two separate incisions for tendon repair [5,6,7,8].

The choice between these surgical approaches remains a topic of debate among orthopedic surgeons, as there is limited consensus regarding their comparative efficacy and associated outcomes. Therefore, a comprehensive comparative analysis of these surgical approaches is necessary to determine the optimal technique for distal biceps tendon rupture repair.

This article presents a comparative analysis of the single-incision technique versus the double-incision technique for distal biceps tendon rupture repair. The study aims to evaluate the clinical outcomes, functional recovery, complications, and patient satisfaction associated with each surgical approach. By critically examining the available evidence and synthesizing the results, this analysis aims to provide valuable insights into the selection of the most appropriate surgical technique for distal biceps tendon rupture.

Understanding the advantages and disadvantages of these surgical approaches is essential for informed decision-making and optimizing patient care. Additionally, identifying the optimal technique can contribute to standardized treatment protocols and enhance clinical outcomes for patients with distal biceps tendon rupture. Through this comparative analysis, we aim to contribute to the existing literature and provide evidence-based recommendations for surgical management strategies in this challenging clinical scenario.

## 2. Materials and Methods

Study Design and Patient Selection: this comparative analysis was conducted at the Orthopedic-Traumatology Clinic II of SCJUPBT Timisoara, Romania, between 2016 and 2022. A total of 48 patients with acute distal biceps tendon rupture and 21 patients with chronic distal tendon rupture were included in the study. The patients were selected based on their diagnosis and eligibility for surgical intervention. Informed consent was obtained from all participants prior to their inclusion in the study.

This study was carried out with the utmost regard for ethical principles and standards governing clinical research.

Informed consent: before participation, all subjects involved in the study were provided with comprehensive information about the study’s objectives, procedures, potential risks, and benefits. Written informed consent was obtained from each participant, ensuring their voluntary participation.

Privacy and confidentiality: personal information of the patients and specific details that could lead to the identification of any participant were kept confidential. Data were anonymized and stored securely, accessible only by the primary research team.

Safety: both surgical approaches discussed in this study are standard in orthopedic surgery. However, careful consideration was given to each patient’s unique medical history and condition before deciding on the appropriate technique. Any adverse events or complications were promptly addressed by the medical team.

Transparency: any potential conflicts of interest among the research team were declared prior to the study’s commencement. The research aimed to provide an unbiased comparative analysis, and any deviations from the protocol or unexpected events were documented transparently.

Ethical approval: this study was conducted following the principles outlined in the Declaration of Helsinki. Ethical approval was secured from the relevant institutional review board or ethics committee before the study’s initiation.

Data integrity: the research team ensured the authenticity and accuracy of the collected data. There was no fabrication, falsification, or inappropriate data manipulation.

The ethical considerations, along with the scientific objectives, underscored the integrity and credibility of this research, ensuring the welfare and rights of all participants were upheld throughout.

Despite its advantage in nerve protection, the dual-incision technique has been associated with a higher incidence of synostosis and heterotopic ossification compared to the single-incision approach. The exact reasons for this increased occurrence are not yet fully understood, but it is believed that the additional incision and disruption of soft tissues may contribute to the formation of these complications.

The distal biceps tendon rupture is a relatively uncommon injury but is most frequently seen in middle-aged men. When it occurs, it can result in significant weakness and functional deficit, especially in forearm supination and elbow flexion. Therefore, surgical repair is often recommended for active individuals.

In the context of surgical approaches to distal biceps tendon repair, the single-incision and double-incision techniques are the most commonly discussed. The Boyd-Anderson technique is one of the older double-incision techniques. Here is a breakdown of the techniques:

Boyd-Anderson Technique (a type of double-incision technique):

developed as a two-incision technique, the Boyd-Anderson approach involves making an anterior incision over the antecubital fossa and a second posterior incision near the radial tuberosity.

The biceps tendon is retrieved through the anterior incision, prepared, and then passed posteriorly to be fixed in the radial tuberosity.

This technique is believed to decrease the risk of certain nerve injuries, especially those to the radial and posterior interosseous nerves.

However, it is associated with higher chances of heterotopic ossification and radioulnar synostosis.

Single-incision technique:

involves an incision in the antecubital fossa where the tendon is retrieved and then reattached.

The single-incision method, depending on the fixation used, has seen an evolution of techniques, including the use of suture anchors, interference screws, and endobuttons.

The main advantage is cosmetic, given there is only one incision.

Main complications can include nerve injuries, particularly the lateral antebrachial cutaneous nerve.

General double-incision technique:

Apart from the Boyd-Anderson technique, other variations of the double-incision technique involve an anterior incision over the antecubital fossa and a second posterolateral elbow incision.

This method was developed to minimize nerve injury.

As previously mentioned, there is a more common occurrence of synostosis and heterotopic ossification with this technique compared to the single-incision approach.

Choosing between these techniques largely depends on the surgeon’s preference and experience, the specific details of the rupture, and patient factors. It is important for patients to discuss with their orthopedic surgeon about the potential risks and benefits of each technique and decide on the best approach tailored to their specific situation.

Follow-up and clinical evaluation: all patients underwent regular follow-up examinations at 1, 2, 6, 12, and 24 months postoperatively. Clinical evaluations were conducted to assess the range of motion (ROM) and isometric strength recovery in comparison to the healthy contralateral side. Nerve injury documentation and assessment were also included in the clinical evaluation. The Disabilities of the Arm, Shoulder, and Hand (DASH) and the Mayo Elbow Performance (MEPS) scores were used to obtain patient-reported outcomes.

The Disabilities of the Arm, Shoulder, and Hand (DASH) score is a validated patient-reported outcome measure that assesses the functional status and disability related to upper limb injuries, including distal biceps tendon rupture. It provides a quantitative assessment of the impact of the injury on various aspects of daily activities, work-related tasks, and social functioning. The DASH score consists of a questionnaire comprising 30 items that cover physical function, symptoms, and social and emotional aspects. Patients rate the severity of their symptoms and the degree of difficulty they experience in performing specific activities on a scale ranging from 0 to 5. A higher score indicates greater disability and functional impairment, while a lower score indicates better functioning and less disability. The Disabilities of the Arm, Shoulder, and Hand (DASH) score is a validated patient-reported outcome measure that assesses the functional status and disability related to upper limb injuries, including distal biceps tendon rupture. It provides a quantitative assessment of the impact of the injury on various aspects of daily activities, work-related tasks, and social functioning. 

The Mayo Elbow Performance Score (MEPS) is a commonly used scoring system to evaluate the functional outcomes and subjective satisfaction of patients following elbow surgeries, including those performed for distal biceps tendon rupture. It provides a standardized and comprehensive assessment of elbow function and pain, allowing clinicians to monitor patient progress and compare outcomes across different treatment approaches. The MEPS consists of both objective and subjective components. The objective component evaluates the range of motion, stability, and strength of the elbow joint, while the subjective component assesses pain, activities of daily living, and patient satisfaction. The score ranges from 0 to 100, with higher scores indicating better elbow function and patient satisfaction. In the objective component, the range of motion of the elbow is measured and compared to the contralateral side or the expected range of motion. Stability is assessed by evaluating the presence of joint laxity or instability. Strength testing includes grip strength and resistance against specific movements, such as flexion and supination. The subjective component of the MEPS focuses on patient-reported outcomes. Patients are asked to rate their pain levels, activities of daily living, and subjective satisfaction with their elbow function. These subjective assessments provide valuable insights into the patient’s perceived functional limitations and overall satisfaction with the surgical outcome.

**Figure 1:**
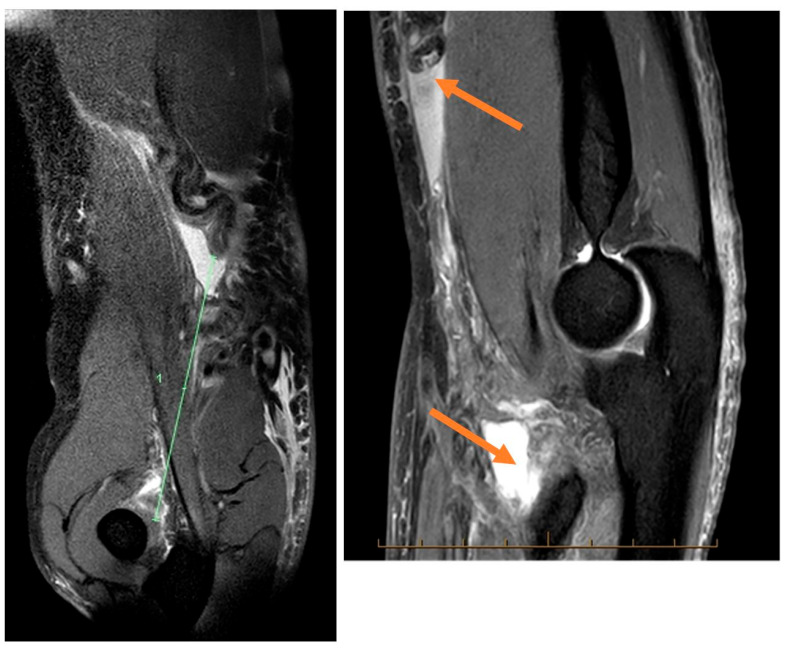
Distal biceps tendon MRI.

Paraclinical investigation: MRI (Figure 1) is the gold standard as far as paraclinical imaging goes, but musculoskeletal ultrasound is also a good option for imaging diagnosis in the case of distal biceps tendon ruptures, X-rays can also be used for imaging diagnosis but are not as efficient as the previous methods.

Preoperative planning and special considerations: during the preoperative planning phase, fixation options were carefully considered. Special instruments such as a 4mm Kerrison rongeur were utilized to widen cortical holes. The combined end button/interference screw technique was employed in certain cases. The surgical approaches were based on the volar Henry approach to the proximal forearm.

Statistical Analysis: Descriptive statistics were used to summarize the patient demographics, surgical techniques employed, and clinical evaluation outcomes. Comparative analysis was conducted between the single-incision and double-incision technique groups using appropriate statistical tests.

Comparative Analysis:

purpose: this method seeks to identify if there are significant differences between two or more groups based on certain variables or outcomes.

Application in this context: a comparative analysis was performed between two groups—those who underwent the single-incision technique and those who underwent the double-incision technique.

Statistical tests: *t*–test was used for comparing two groups include the *t*-test (for normally distributed data).

The distal biceps tendon rupture is an injury that often requires surgical intervention to restore arm strength and function. Two primary techniques have been widely adopted in orthopedic surgery: the single-incision and the double-incision techniques.

Single-incision technique:

this method utilizes a singular incision over the antecubital fossa. The tendon is then retrieved and reattached, often using suture anchors or interference screws.

Advantages: it leaves a smaller surgical scar and often has a faster initial recovery period.

Drawbacks: there is a potential risk of injury to the lateral antebrachial cutaneous nerve. In some cases, depending on the method of fixation, there might also be a risk of heterotopic ossification.

Double-incision technique:

the technique involves two incisions: an anterior one in the antecubital fossa and a second one made posteriorly near the radial tuberosity.

Advantages: direct visualization of the radial tuberosity allows for a more accurate reattachment. Moreover, there is a potentially reduced risk of certain nerve injuries.

Drawbacks: there is a higher risk of radioulnar synostosis and a more prominent scar due to two incisions.

Surgical complications: the study documented and analyzed both major and minor complications associated with the surgical approaches. Major complications included proximal radio-ulnar synostosis, posterior interosseous nerve (PIN) palsy, and atraumatic tendon re-rupture. Minor complications included intermittent pain, range of motion deficiency, isometric strength deficiency, lateral antebrachial cutaneous nerve (LACBN) injury, and infection.

Data analysis: the data collected from the clinical evaluations and patient-reported outcomes were analyzed using appropriate statistical methods, including paired *t*-tests and chi-square tests, as applicable.

The specific surgical techniques, patient follow-up, clinical evaluation methods, and statistical analysis mentioned above were employed to comprehensively compare the outcomes and complications associated with the single-incision technique versus the double-incision technique for distal biceps tendon rupture repair in this study.

## 3. Results

**Figure 2:**
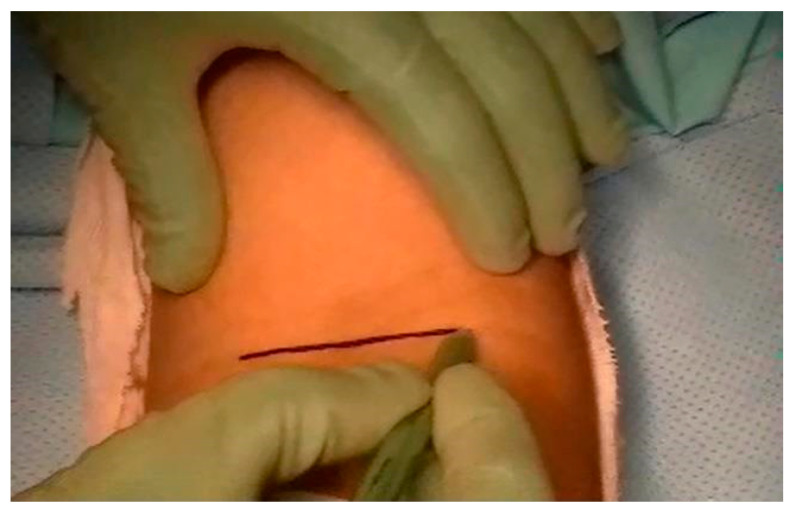
Single-incision technique approach.

**Figure 3:**
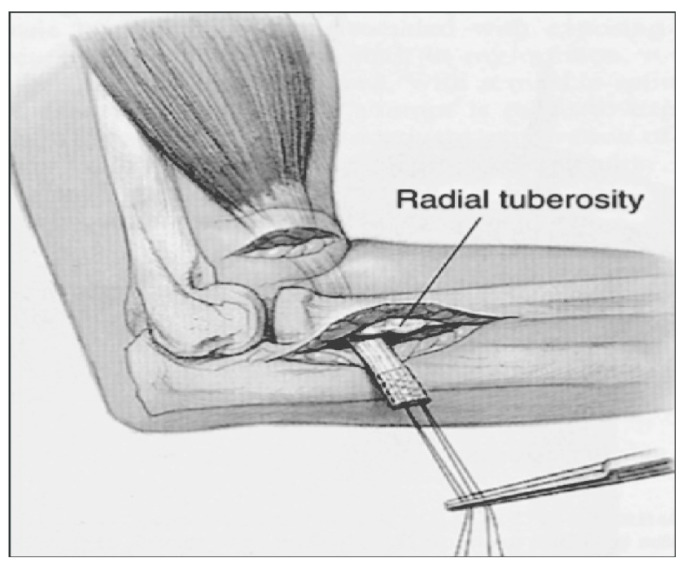
Double-incision technique approach.

Among the study participants, 45 cases underwent the single-incision technique (Figure 2) with the distal biceps repair system–endobutton and screw, while 24 patients were treated with the modified double-incision technique. The modified double-incision technique (Figure 3) involved the Morrey-modified approach, Krackow sutures, and drill holes. The surgical procedures were performed by experienced orthopedic surgeons at the clinic.

### 3.1. Anterior Single-Incision Technique

Incision from antecubital fossa: the most common complication is the injury to the lateral antebrachial cutaneous nerve. Sometimes the radial nerve or posterior interosseous nerve might be damaged.

Less common heterotopic ossification and synostosis compares to dual-incision technique.

Ethical considerations: this study was conducted in accordance with the ethical principles outlined in the Declaration of Helsinki. Ethical approval was obtained from the Pius Brinzeu County Hospital-Review Board prior to the commencement of the study.

### 3.2. Dual-Incision Technique

The method is developed to avoid injury to a radial nerve or posterior interosseous nerve. It includes a smaller anterior incision over the antecubital fossa and a second posterolateral elbow incision.

**Figure 4:**
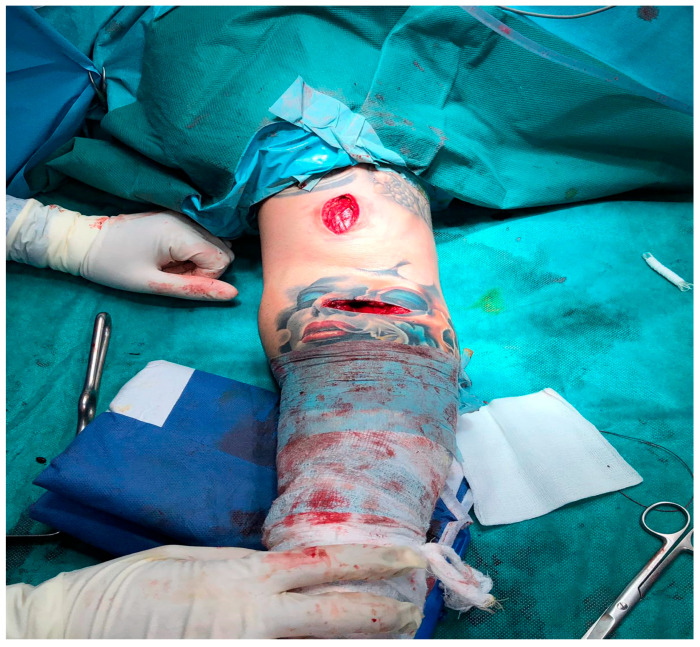
One incision tehnique and additional incision.

The dual-incision technique is more common to develop synostosis and heterotopic ossification than the single incision approach (Figure 4).

### 3.3. Rehabilitation Goals after Surgery (Figure 5)

Phase I (up to 2 weeks after surgery)-protection of healing repair (Splint: Your elbow will be immobilized at 90° in a splint for 10–14 days with forearm in neutral).

Phase II (2–4 weeks after surgery)-protect repair; avoid overstressing the fixation site; begin to restore motion. 

Phase III (5–12 weeks after surgery)-achieve full elbow motion; adherence to home exercise program (HEP).

Phase IV (begin after meeting Phase III criteria, usually 12 weeks after surgery)-normal multi-planar high velocity movements without side to side differences or compensations; normal strength without side to side differences or compensations; adherence to HEP.

The subjects were enrolled ina follow-up regimen spanning intervals of 1, 2, 6, 12, and 24 months. The follow-up involved a comprehensive clinical assessment, encompassing the measurement of range of motion (ROM) and isometric strength recovery in comparison to the corresponding healthy contralateral side. Additionally, documentation of any nerve injuries was carried out.

Among the cohort of patients who underwent the single incision technique, 36 patients exhibited full range of motion with no resultant complications. These 36 patients were subjected to evaluations at the aforementioned time points: 1, 3, 6, 12, and 24 months.

In the group of patients treated with the single incision method, 9 patients experienced intermittent pain and demonstrated deficits in range of motion (ROM) below 30° in flexion/extension and pronation/supination. 

These patients also exhibited deficiencies in isometric flexion strength, with deficits below 30%, and isometric supination strength, with deficits below 60%. Furthermore, one patient within this group exhibited an injury to the lateral antebrachial cutaneous nerve (LACBN).

Among the 24 patients who were subjected to the double incision technique, 14 patients demonstrated unimpeded complete range of motion without experiencing any ensuing complications. Additionally, 10 of these patients experienced significant postoperative complications, encompassing 6 instances of proximal radio-ulnar synostosis and 4 cases of posterior interosseous nerve (PIN) palsy.

In 5 instances where the single-incision approach was employed as a treatment modality, the patients presented with a chronic tendon rupture of approximately 2 months duration. Consequently, during the surgical intervention, the decision was made to execute an additional incision to locate the retracted tendon, followed by the implementation of fixation using a screw and a button.

Among the total study participants, 45 cases underwent the anterior single-incision technique utilizing the distal biceps repair system–endobutton and screw. Conversely, 24 patients were treated with the modified dual-incision technique, encompassing the Morrey-modified approach, Krackow sutures, and drill holes. 

Notably, these surgical interventions were executed by seasoned orthopedic surgeons stationed at our clinic. Post-surgery, patients were subjected to a systematic rehabilitation process with distinct phases, each with its unique objectives. 

These phases ranged from immediate post-op care to a more extended recovery spanning 12 weeks. To gauge the efficacy and outcomes of these techniques, subjects were routinely monitored at set intervals of 1, 2, 6, 12, and 24 months. 

These follow-ups were comprehensive, measuring metrics like range of motion (ROM) and isometric strength, juxtaposed against the unaffected contralateral side. Crucially, instances of nerve injuries were meticulously documented.

## 4. Discussion

Delving into the specifics of the anterior single-incision technique, it is noteworthy that the most prevalent complication involved injury to the lateral antebrachial cutaneous nerve, with potential damage to the radial or posterior interosseous nerve. This technique had a lower incidence of heterotopic ossification and synostosis relative to the dual-incision approach.

On the other hand, the dual-incision technique was formulated with a primary focus on mitigating nerve injury risks. However, it bears its own set of challenges, including a heightened propensity for synostosis and heterotopic ossification compared to its single-incision counterpart.

Our ethical commitments were unwavering throughout this research. The study conformed scrupulously to the ethical principles encapsulated in the Declaration of Helsinki, with the Pius Brinzeu county hospital-review board bestowing their ethical approval before the study’s onset.

Surgical techniques: among the study participants, 45 cases underwent the single-incision technique with the distal biceps repair system, while 24 patients were treated with the modified double-incision technique. The modified double-incision technique involved the Morrey-modified approach, Krackow sutures, and drill holes. The surgical procedures were performed by experienced orthopedic surgeons at the clinic.

Anterior single-incision technique: the anterior single-incision technique for distal biceps tendon rupture repair involves a single incision made from the antecubital fossa. This approach offers certain advantages, including simplicity and a lower risk of complications compared to the dual-incision technique. However, it is important to note that this technique is not without its own set of complications.

The most frequently encountered complication associated with the anterior single-incision technique is injury to the lateral antebrachial cutaneous nerve. This nerve runs in close proximity to the surgical site and is susceptible to inadvertent damage during the procedure. Additionally, there is a risk of injury to the radial nerve or posterior interosseous nerve, although this is less common compared to the dual-incision technique.

In terms of postoperative complications, the anterior single-incision technique has demonstrated a lower incidence of heterotopic ossification and synostosis compared to the dual-incision technique. Heterotopic ossification refers to the abnormal formation of bone in soft tissue, while synostosis refers to the fusion of adjacent bones. These complications can restrict joint movement and potentially compromise overall patient outcomes.

Dual-incision technique: the dual-incision technique was developed as an alternative to the single-incision technique to specifically mitigate the risk of injuring the radial nerve or posterior interosseous nerve during distal biceps tendon repair. This technique involves making a smaller anterior incision over the antecubital fossa, as well as a second posterolateral elbow incision.

The comparative analysis of surgical approaches for distal biceps tendon rupture has been a subject of considerable interest within the field of orthopedics. This study aimed to elucidate the key differences between the single-incision and double-incision techniques with regard to various clinical parameters, including complications, re-ruptures, flexion, supination strength, and endurance.

Our findings align with previous research, demonstrating that there is limited divergence between these two approaches in terms of the aforementioned parameters. The outcomes suggest that both the single-incision and double-incision methods can yield comparable results in managing distal biceps tendon ruptures, emphasizing the efficacy of surgical intervention for this type of injury.

Notably, this study contributes to the growing body of evidence that supports the single-incision technique as a viable treatment option. While our results do not show significant discrepancies in complications, re-rupture rates, or measures of flexion and supination strength and endurance, an important revelation emerged concerning the fidelity of footprint position. The double-incision approach, as demonstrated in recent studies, more closely reproduces the anatomical footprint, potentially contributing to improved long-term outcomes in terms of stability and functional restoration [8,9,10,11,12].

The significance of anatomical restoration cannot be overstated, as it has been associated with superior clinical outcomes and reduced likelihood of long-term sequelae [12,13,14,15]. The ability of the double-incision technique to achieve a more precise footprint alignment could be crucial in cases where meticulous reconstruction is essential, such as in high-demand individuals or athletes.

Despite these intriguing findings, it is essential to acknowledge the limitations of our study. The sample size might have influenced our ability to detect subtle differences in certain parameters. Additionally, longer-term follow-up is necessary to fully assess the impact of footprint accuracy on patient outcomes, including the incidence of late complications and the rate of return to pre-injury levels of activity [15,16].

The distinct clinical features typically associated with an acute rupture of the distal biceps render additional imaging modalities such as radiographs, MRI (was it uniquely performed under Flexed Abducted Supinated View), or ultrasound unnecessary for diagnostic purposes. In recent decades, surgical intervention for this specific type of lesion has consistently demonstrated superior functional outcomes in comparison to conservative management strategies. A notable study by Baker et al. conducted a rigorous comparative analysis, revealing compelling evidence of diminished supination strength by 55% and a substantial reduction in supination endurance by 86% when employing the nonoperative approach as opposed to controls [1,16,17,18,19,20,21,22].

These findings underscore the pivotal role of surgical repair in optimizing the functional recovery of individuals with distal biceps ruptures. The disparities observed in supination strength and endurance emphasize the potential limitations of conservative measures, underlining the importance of considering surgical intervention as the preferred treatment strategy. The implications of such decreased functional capabilities are substantial, particularly in the context of tasks that rely heavily on supination, such as lifting, gripping, and manipulating objects in daily life [19,20,21,22].

It is essential to recognize that while surgical repair has proven advantageous, the specific choice of operative technique is a subject of ongoing exploration within the medical community. This includes the investigation of the single-incision versus double-incision approaches, as this comparative analysis aims to provide valuable insights into the optimal surgical approach for distal biceps tendon ruptures. The challenge lies in achieving a balance between effective restoration of function and minimizing potential complications associated with surgery.

Further research, including larger studies with extended follow-up periods, is warranted to refine our understanding of the long-term outcomes and potential advantages of different surgical techniques. These efforts will ultimately inform evidence-based clinical decision-making, tailoring treatment strategies to individual patient characteristics and optimizing their chances for successful functional recovery and improved quality of life.

## 5. Conclusions

In conclusion, while the anterior single-incision technique for distal biceps tendon rupture repair offers the advantage of a simpler procedure and a reduced risk of certain complications, such as synostosis and heterotopic ossification, it is important to carefully consider and manage the risk of injury to the lateral antebrachial cutaneous nerve, as well as the potential for damage to the radial nerve or posterior interosseous nerve. 

Conversely, the dual-incision technique aims to avoid nerve injury but carries a higher risk of synostosis and heterotopic ossification. 

The choice between these surgical approaches should be individualized based on patient-specific factors and careful consideration of the associated benefits and risks. Further research and comparative studies are warranted to provide additional insights into the long-term outcomes and complications of both techniques.

Recent clinical investigations have uncovered limited disparities between the single-incision and double-incision techniques regarding complications, re-ruptures, and measures of flexion and supination strength and endurance. Nevertheless, emerging evidence highlights that the double-incision approach more accurately replicates the footprint position in comparison to the 1-incision method. This finding underscores the growing importance of anatomical fidelity in optimizing surgical outcomes for distal biceps repair.

## Figures and Tables

**Figure 5:**
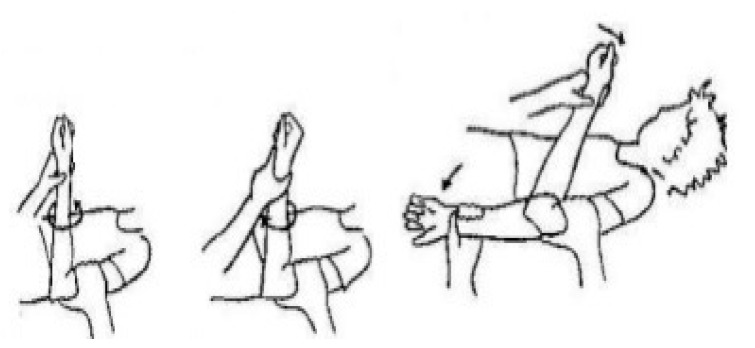
Rehabilitation goals after surgery.

## Data Availability

Not applicable.
